# A Rare Case of Cold Agglutinin Syndrome Associated With Legionella Pneumonia

**DOI:** 10.7759/cureus.41310

**Published:** 2023-07-03

**Authors:** Khalid Shakfeh, Fatimat Shotande, Candice Mateja

**Affiliations:** 1 Internal Medicine, University of South Florida Morsani College of Medicine, Tampa, USA; 2 Internal Medicine-Pediatrics, University of South Florida, Tampa, USA

**Keywords:** microspherocytosis, legionella infection, immune hemolytic anemia, cold agglutinin disease, legionella

## Abstract

Cold agglutinin syndrome (CAS) is a rare subset of autoimmune hemolytic anemia (AIHA) and can be classified as either primary or secondary. Secondary cold agglutinin disease has been associated with both viral and bacterial pathogens with the most common bacterial pathogen being *Mycoplasma pneumoniae*. *Legionella *pneumonia is a well-known causative agent for community-acquired pneumonia that can lead to a severe disease requiring hospitalization that is rarely associated with AIHA. We highlight the importance of recognizing *Legionella* pneumonia as a causative pathogen for CAS.

## Introduction

*Legionella* is a pathogenic gram-negative intracellular bacteria found in freshwater environments. *Legionella* pneumonia can present as a community-acquired pneumonia, and as of 2017 was reported to be a cause of pneumonia in 2.29 per 100,000 persons. Although there has been a significant increase in the reported incidence of *Legionella* pneumonia over the past two decades, it is still considered to be highly underreported [1]. *Legionella* is relatively indistinguishable from other pneumonia, requiring high clinical suspicion. Characteristics unique to *Legionella* pneumonia include gastrointestinal symptoms, relative bradycardia, neurological manifestation, hyponatremia, and elevated liver enzymes. Treatment generally consists of azithromycin or levofloxacin [2].

Autoimmune hemolytic anemia (AIHA) is a hemolytic process caused by the body’s immune system targeting red blood cell antigens and is categorized as either warm or cold [3]. Cold agglutinin antibodies are associated with IgM and activate at 3-4 degrees Celsius [4].

Cold agglutinin syndrome (CAS) is primary or secondary. Secondary cold agglutinin is associated with infections, autoimmune disease, and lymphoma, whereas primary is often idiopathic. Treatment for secondary CAS involves treating the underlying cause and supportive care. The most commonly associated infective agents leading to CAS include *Mycoplasma pneumoniae* and the Ebstein-Barr virus [5].

## Case presentation

A healthy 40-year-old gentleman presented to the emergency department with two days of dyspnea and fevers. His home pulse oximeter measured 90% on ambient air, which prompted his visit to the emergency department. He had abdominal discomfort and noticed he was pale but denied diarrhea and bloody bowel movements. He denied drug use, supplements, recent travel, or sick contacts. He works in a warehouse without any known toxic exposures but had AC exposure.

On presentation, he was oriented and in no distress. He was normotensive, afebrile, tachycardic to 105 beats per minute, and saturating 92% on 2L of oxygen. There were rales on the auscultation of his right lung, and he did not demonstrate any increased work of breathing. His abdomen was soft and nontender, without organomegaly. He exhibited mucosal pallor and pale skin. The remainder of his exam was unremarkable.

**Figure 1:**
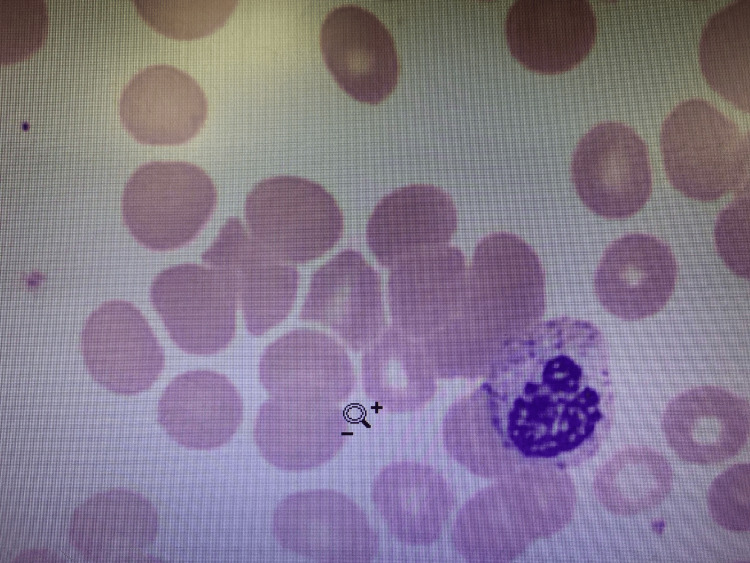
Peripheral smear demonstrating microspherocytosis with agglutination

**Figure 2:**
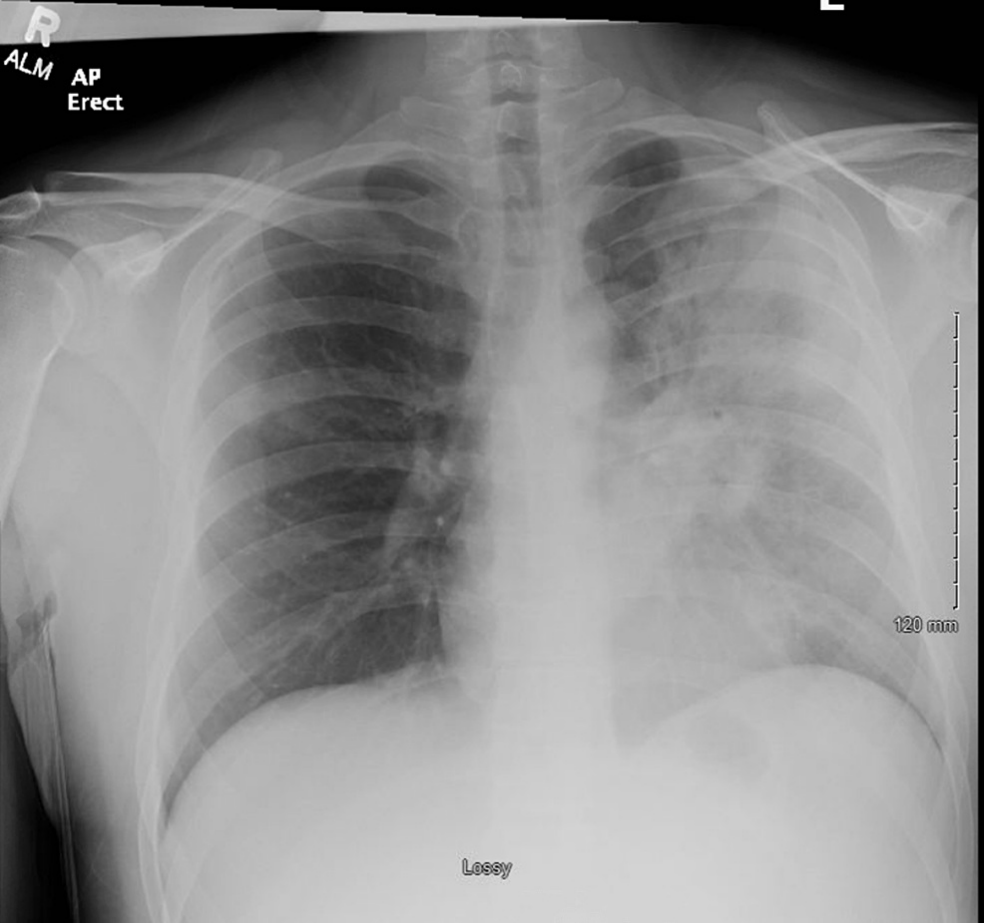
Chest x-ray demonstrating multilobar pneumonia

Initial laboratory studies are displayed in Table 1. A peripheral smear demonstrated red blood cell agglutination (Figure 1). A urine *Legionella* antigen was positive; blood cultures and a respiratory pathogen panel (including M. pneumonia and EBV) were negative. His chest radiograph showed multilobar pneumonia (Figure 2). Baseline laboratory values six months ago during a primary care visit were all within normal limits.

**Table 1:** Labs on the day of admission WBC = white blood cells; RBC = red blood cells; MCV = mean corpuscular volume; BUN = blood urea nitrogen; AST = aspartate aminotransferase; ALT = alanine transaminase; LDH = lactate dehydrogenase; TIBC = total iron binding capacity

	Result	Reference Range
WBC (10^9 cells/L)	16	4.5-11
RBC (10^12 cells/L)	2	4.3-5.9
Hemoglobin (g/dL)	7	13.5-17.5
MCV (fl)	97	80-100
Platelets (g/dL)	208	150-400
% Neutrophils	53	50-70
% Bands	37	2.0-6.0
% Lymphocytes	2	20-40
%Monocytes	4	2.0-8.0
%Eosinophils	1	1.0-3.0
%Atypical lymps	2	0
Sodium (mEq/L)	129	135-145
Potassium (mEq/L)	3	3.5-5.5
Chloride (mEq/L)	97	96-106
Bicarbonate (mEq/L)	26	22-26
BUN (mEq/dL)	19	6-24
Creatinine (mg/dL)	1	0.6-1.3
Glucose (mg/dL)	124	70-140
Total bilirubin (mg/dL)	6	0.1-1.2
Direct Bilirubin (mg/dL)	2	0-0.3
AST (U/L)	270	10-40
ALT (U/L)	64	7-56
LDH (U/L)	1256	140-280
Haptoglobin (mg/dL)	<8	41-165
Ferritin (ng/mL)	28911.4	24-336
Iron (mcg/dL)	58	60-170
TIBC (mcg/dL)	160	250-450
% Reticulocyte	1	0.5-2.5
Cold Agglutinins	1:320	<1:64
Direct Coombs	IgG -, C3d +	Negative

The patient was admitted to the hospital and started on empiric treatment for community acquired pneumonia. Following fluid resuscitation, his hemoglobin decreased to 5.5 g/dL. A type and cross were drawn and sent to a specialized facility due to the degree of agglutination. He received warmed trauma blood at 37 degrees Celsius and was kept warm via blankets and ambient heating. After identifying *Legionella* as the causative organism, antibiotics were narrowed to azithromycin. By hospital day 2, his laboratory studies demonstrated cessation of hemolysis. His hemoglobin remained at 7.5 g/dL, his indirect bilirubin dropped to 2 mg/dL, and haptoglobin levels increased to 28 mg/dL. With the resolution of his hemolysis and hypoxia, he was transitioned to oral antibiotics azithromycin. He was monitored for one additional day given the degree of his anemia, and ultimately discharged on hospital day 3. Hematology-Oncology was consulted and recommended outpatient follow-up for further workup.

## Discussion

This patient was ultimately diagnosed with *Legionella* pneumonia complicated by CAS. While *Mycoplasma* species are frequently associated with CAS, there is a lower association with *Legionella*. There are only five case reports published at the time of this submission of an AIHA associated with *Legionella*, and only one published associated with cold agglutinin [6-8]. This raises the possibility of underdiagnosis of AIHA secondary to *Legionella*.

In patients with new anemia in the setting of *Legionella*, a further workup including LDH, haptoglobin, and a peripheral smear would be reasonable. If there is evidence of hemolysis on blood work, with concurrent microspherocytosis, a Coombs test should be ordered. After the resolution of any acute process that is considered the source of CAS, patients should follow up with hematology to ensure resolution or additional workup.

Treatment for CAS is unique compared to other autoimmune processes. While steroids are generally favored for an autoimmune process, they do not seem to significantly affect the disease course of CAS. Rather, supportive care by keeping the patient warm is the first line of treatment. Rituximab or cyclophosphamide can be adjuncts in the case of refractory symptoms. In our patient, supportive care while treating the underlying cause of his CAS was sufficient. While data with *Legionella* and CAS is sparse, correlations can be drawn from other atypical pneumonia that triggers CAS, most notably *M. pneumoniae*. Various case reports have demonstrated that the hemolytic process resolves with the resolution of the infection in conjunction with supportive care, as demonstrated by Eldin et al. and Kanagevelu et al. [9,10].

Considering the sparsity of data of this unique disease process, there is limited guidance on antibiotic selection and duration. While general practice and recommendations in community-acquired pneumonia suggest a transition to oral antibiotics when patients demonstrate clinical response followed by discharge [11], we elected to continue observation for at least one additional day while on oral antibiotics considering the degree of his anemia. This addition to the literature highlights the importance of having a high clinical suspicion of *Legionella* pneumonia and its association with cold agglutinin AIHA.

## Conclusions

CAS should be considered on the differential when a patient presents with atypical pneumonia and new onset anemia. While this is generally associated with *Mycoplasma *species, *Legionella *pneumonia should be considered as well. In addition to the appropriate selection of antibiotics, supportive measures, specifically keeping the patient warm, should be implemented. Warmed blood transfusions should also be considered depending on the patient's degree of anemia.
